# Reflective Bistable Chiral Splay Nematic Liquid Crystal for Low-Power Heat Sensor

**DOI:** 10.3390/s20215937

**Published:** 2020-10-22

**Authors:** Chao Ping Chen, Chul Gyu Jhun

**Affiliations:** 1 Smart Display Lab, Department of Electronic Engineering, Shanghai Jiao Tong University, Shanghai 200240, China; ccp@sjtu.edu.cn; 2 Department of Display Engineering, Hoseo University, Asan, Chungnam 336-795, Korea

**Keywords:** liquid crystal, bistable chiral splay nematic, low-power, heat sensor

## Abstract

The memory effect of the bistable liquid crystal mode is able to maintain the display information for a long time. The splay state and π twist states are used as the memory states of the bistable chiral splay nematic (BCSN) mode. The transition time from the π twist state to the splay state is sensitive to the temperature. In this paper, for the heat sensor application, the reflective structure of the BCSN mode has been studied by the Jones matrix method. In experiments, the measured contrast ratio can be over 200 with a minimal reflective structure including a single polarizer and a reflector.

## 1. Introduction

With the recent development of refrigeration and freezing technology, a variety of agricultural and marine products are readily available to consumers at a relatively low cost. However, as the travel distance for distribution increases and the distribution time becomes longer, transparency of the distribution process is required for the products or collected articles circulated by refrigerated or frozen storage. There are a variety of methods for tracking the distribution history such as tracking the temperature of the workplace in the production stage, tracking the temperature of the loading car of the refrigerated vehicle. However, there is also a problem in that, when the loading and unloading operations are performed at each distribution stage, even if they are exposed to the outside for a long time, the temperature change cannot be accurately reflected. In other words, it is necessary to keep the temperature of refrigeration or freezing during circulation at an optimal temperature depending on each of them. Even though the time for exposure to the outside environment in the circulation process is prolonged and the temperature of the products is increased, if refrigerated or frozen, consumers cannot confirm it. In addition, this tracking method has the disadvantage that it cannot be confirmed by the end-user, since only the production and distribution companies provide temperature changes. From this point of view, there is a need for a heat sensor [1,2,3,4] that can be confirmed by the consumer.

Bistable liquid crystal devices [5,6,7,8,9,10,11], which are suitable for the price tag, the e-book reader, signboard, and so on, have many advantages. The memory effect, among other things, which is able to maintain the display information permanently, can reduce the power consumption. In addition, if the memory mode is designed to be a reflection type, for which the backlight source is not necessary, the power consumption is minimized and it can be used quasi-permanently [12]. In this paper, we demonstrate a low-power heat sensor based on a bistable chiral splay nematic (BCSN) liquid crystal device.

## 2. Operational Principle

### 2.1. Swtiching Process

**Figure 1:**
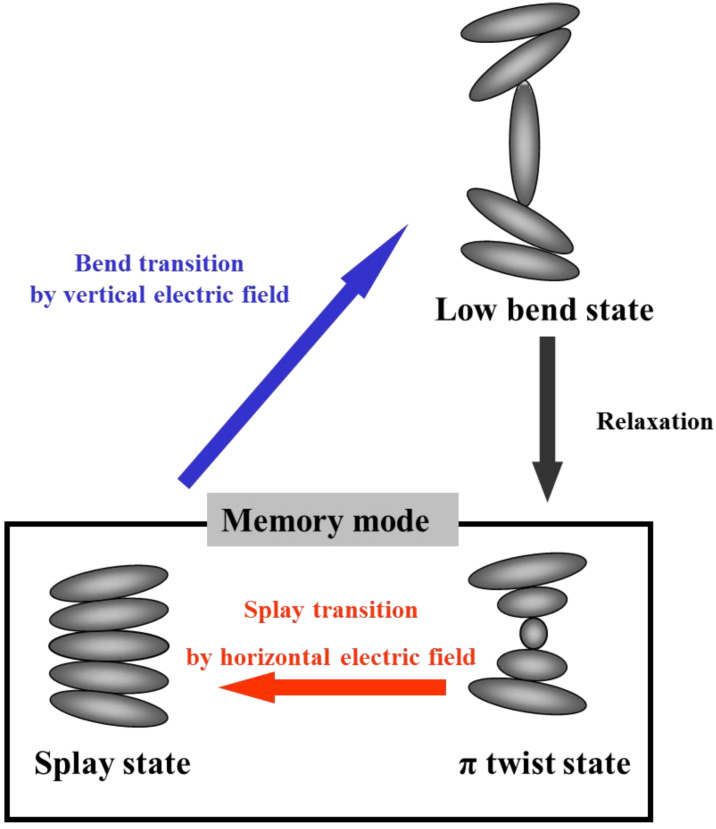
Phase transition of bistable chiral splay nematic (BCSN) mode. When the vertical electric field is applied to the splay state, it is switched to the bend state represented by the blue arrow. As the applied field is removed, the bend state is relaxed into the π twist state represented by the black arrow. By the horizontal electric field, the π twist state is switched into the splay state represented by the red arrow.

The switching process between the splay state and the twist state, which is the memory state in the bistable chiral splay nematic (BCSN) mode, is shown in Figure 1 [13]. When the vertical electric field is applied to the splay state, it is switched to the bend state by dielectric interaction with electric the field. As the applied field is removed, the bend state is relaxed into the π twist state because of the topological equivalence. By the horizontal electric field, the π twist state is switched into the splay state. For the low-power sensor application, instead of relying on the electric field, it is more desirable to trigger the phase transition via the heat.

### 2.2. Bistable Curve

The bistable properties the BCSN mode can be characterized by the bistable curves [14]. We have calculated the bistable curves in terms of the free energy *F* for the nematic liquid crystal cell in one dimension, which is defined in polar coordinate as

(1) $$F=\int_{0}^{d}\left\{\frac{f\left(\theta \right)}{2}{\left(\frac{d\theta }{dz}\right)}^{2}+\frac{g\left(\theta \right)}{2}{\left(\frac{d\varphi }{dz}\right)}^{2}+e\left(\theta \right)\left(\frac{d\varphi }{dz}\right)+\frac{K_{22}q_{0}^{2}}{2}\right\}dz+F_{s}$$

where

(2) $$f\left(\theta \right)=K_{11}sin^{2}\theta +K_{33}cos^{2}\theta$$

(3) $$g\left(\theta \right)=\left(K_{22}sin^{2}\theta +K_{33}cos^{2}\theta \right)sin^{2}\theta$$

(4) $$e\left(\theta \right)=-K_{22}q_{0}sin^{2}\theta$$

*θ* and *φ* are the polar and azimuthal angles of liquid crystal directors, respectively. *K*_11_, *K*_22_, and *K*_33_ are the splay, twist and bend elastic constants of the liquid crystal, respectively, and *q*_0_ is the chirality related to the pitch *P*_0_ by *q*_0_ = 2*π*/*P*_0_. Under one constant approximation (*K*_11_ = *K*_22_ = *K*_33_ = *K*), the free energy could be simplified as

(5) $$F=\int_{0}^{d}\left\{\frac{K}{2}{\left(\frac{d\theta }{dz}\right)}^{2}+\frac{K}{2}sin^{2}\theta {\left(\frac{d\varphi }{dz}\right)}^{2}-Kq_{0}sin^{2}\theta \left(\frac{d\varphi }{dz}\right)+\frac{Kq_{0}^{2}}{2}\right\}dz+F_{s}$$

The surface anchoring energy *F_s_* is taken into account by the Rapini–Papoular surface potential [15]

(6) $$F_{s}=\frac{1}{2}A_{p}sin^{2}\left(\theta -\theta_{0}\right)+\frac{1}{2}A_{a}sin^{2}\left(\varphi -\varphi_{0}\right)$$

**Figure 2:**
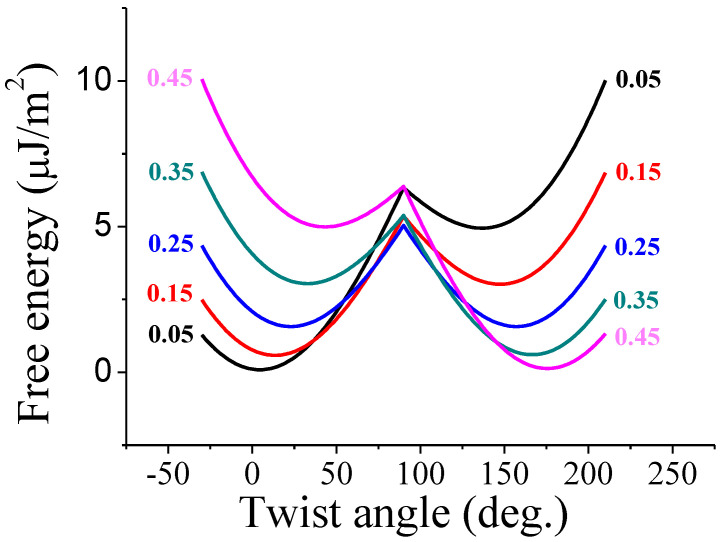
Bistable curves with respect to the different *d*/*p* ratios. The approximated elastic constant is 10 pN and both the polar and azimuthal anchoring coefficients are 1 × 10^−5^ J/m^2^. The cellgap and pretilt angle are in turn 6 μm and 5°.

Here, *A_p_* and *A_a_* are the polar and azimuthal anchoring coefficients, respectively. Using Equation (5) and Equation (6), and given the director profiles, the variations of the Gibbs free energy per unit area with respect to the twist angle, namely, the bistable curve, can be obtained by a straightforward calculation. The bistable curves calculated with respect to the cellgap-to-pitch (*d*/*p*) ratio are shown in Figure 2. In our calculation, the approximated elastic constant is 10 pN and both the polar and azimuthal anchoring coefficients are 1 × 10^−5^ J/m^2^. The cellgap and pretilt angle are in turn 6 μm and 5°. When the *d*/*p* ratio is 0.25, two local minimums of free energy for the two stable states are theoretically identical. Therefore, with the *d*/*p* ratio of 0.25, ideal bistable properties can be achieved. If the *d*/*p* ratio is less than 0.25, asymmetric bistable curve, in which the energy of the splay state is lower than that of the π twist state, is obtained. In this case, the memory time of the splay state will be infinite, while the π twist state is not.

### 2.3. Phase Transition

**Figure 3:**
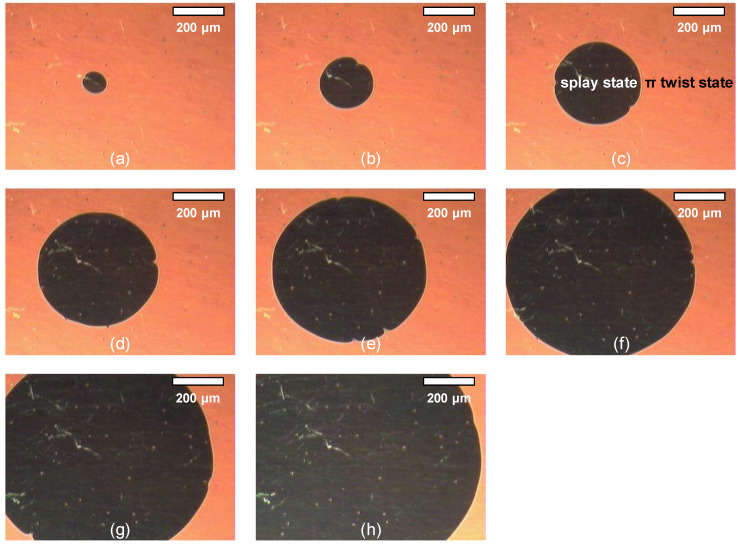
Phase transition from the initial twist state to the splay state observed at (**a**) 6 s, (**b**) 9 s, (**c**) 12 s, (**d**) 15 s, (**e**) 18 s, (**f**) 21 s, (**g**) 24 s, and (**h**) 27 s.

With the *d*/*p* ratio being less than 0.25, the π twist state is replaced by the splay state accompanying the motion of the disclination line, which is the boundary of two domains, starting from the defects or pixel boundaries. In our experiment, the liquid crystal material is ZLI-2293 with a thickness of 6 μm and a *d*/*p* ratio of 0.2. Figure 3 shows the phase transition from the initial twist state to the splay state. As time goes on, the domain of the splay state gradually penetrates into the domain of the twist state.

### 2.4. Transition Velocity

The disclination line is the boundary of the splay state. The moving speed of the disclination line is influenced by the liquid crystal properties and has an important influence on the transition from the twist state to the splay state. The velocity of the disclination line is derived from the equilibrium relationship between the loss rate of free energy and dissipation [16]. By creating an identity, which describes the balance of the time rate of the free energy *F* stored within a space and the dissipated energy *D* in the same space, a simple model is derived as follows [16]:

(7) F+D=0

If the flow effect is neglected, the dissipation *D* can easily be given by the volume integral of the viscous torques acting on the liquid crystal director *n*

(8) $$D=\gamma \int {\dot{n}}^{2}dV$$

where *γ* is the rotational viscosity and $\dot{n}$ is the time derivative of director *n*. Corresponding to *D*, *F* becomes

(9) $$F=-\gamma \int {\dot{n}}^{2}dV=-\gamma v^{2}\int {\left(\frac{\partial \theta }{\delta x}\right)}^{2}dV$$

where the motion of the disclination line is along the *x*-axis. The integral term in *F* is assumed to be the damping factor *G* [17], and the damping factor is related to the device parameter by

(10) $$G=\frac{\pi }{4}ln\left(\frac{d}{2a}\right)$$

here, *a* (=250 nm) is the diameter of the disclination core and *d* is the cellgap. From the above equations, we could finally arrive at the velocity of movement of disclination line [17]

(11) $$v=\frac{\Delta F}{\gamma G}$$

to be applicable as the heat sensor, we choose the *d*/*p* ratio of 0.2 to make the splay state more stable than the other stable state and to have appropriate velocity. The transition status is dependent on the time and temperature due to the temperature dependence of the rotational viscosity value. That is, when the π twist state is subject to heat at a certain temperature for a certain time, the velocity of transition increases.

### 2.5. Temperature Measurement

**Figure 4:**
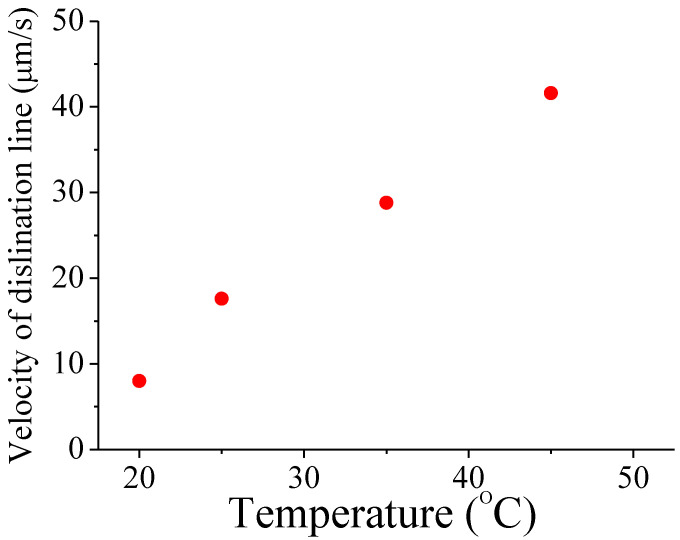
Measured velocity of the disclination line with respect to the temperature.

The viscosity of a liquid crystal depends on the temperature [18]. As the temperature increases, the viscosity decreases. Therefore, it can be predicted that as the temperature increases, the moving speed of the disclination line increases for the splay transition. To verify the temperature dependence of the velocity of the disclination line, liquid crystal cells with 6 μm thickness are prepared. This BCSN mode is fabricated with a liquid crystal material of BHR71200-100 (Linktecs). The *d*/*p* ratio is 0.2. The transition characteristics of the fabricated device according to temperature is measured. As shown in Figure 3, the movement of disclination line can be captured under a camera with a built-in calibrated ruler. The velocity of the disclination line is determined as the distance of its movement per unit time. Temperature from 20 °C to 45 °C is controlled by a hot stage (Mettler Toledo). The measured temperature effects on the velocity of the disclination line is shown in Figure 4. The velocity is 8 μm/s at 20 °C. It increases linearly with temperature and becomes 41.6 μm/s at 45 °C.

## 3. Optical Structure of Reflective BCSN Device

### 3.1. Reflective Structure

**Figure 5:**
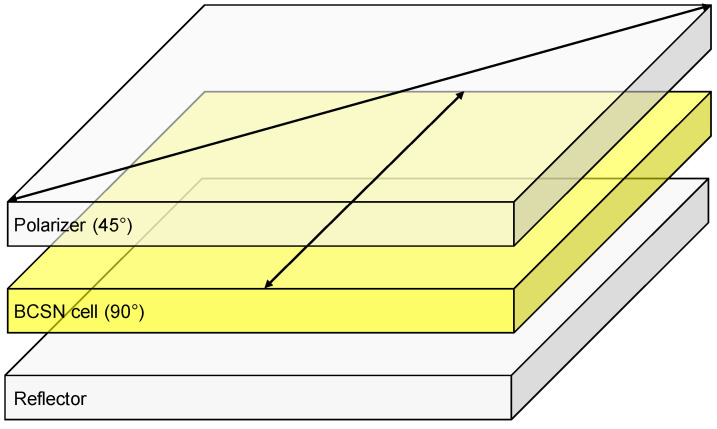
Optical structure of the reflective BCSN mode. The transmission axis of the polarizer and the rubbing direction of the liquid crystal cell are intersected by 45° so that a dark state could be obtained at the splay state.

The desired low-power heat sensor should be designed with a reflective structure that does not require the light source. In order to ensure price competitiveness, the minimal reflective structure, consisting of a single polarizer, a BCSN cell and a reflector, is adopted. The transmission axis of the polarizer and the rubbing direction of the BCSN cell are intersected by 45° such that the dark and bright states could be obtained at the splay state and twist state, respectively, as shown in Figure 5.

### 3.2. Reflectance

The optical structure of the proposed BCSN heat sensor is designed to realize the maximum contrast ratio (CR) with the splay state and the π twist state. Reflectance *R* of the splay and π twist states can be calculated by the Jones matrix method [19,20,21]. The reflectance of the two states can be calculated using Equation (12)

(12) $$R=\;\left|\left(cos\alpha \;sin\alpha \right)\cdot M_{T}\cdot M\left(\begin{array}{c} cos\alpha \\ sin\alpha \end{array}\right)\right|$$

where

(13) $$M=\left|\begin{array}{cc} cos\phi & -sin\phi \\ sin\phi & cos\phi \end{array}\right|\left|\begin{array}{cc} cosX-i\frac{\gamma }{2}\frac{sinX}{X} & \phi \frac{sinX}{X}\\ -\phi \frac{sinX}{X} & cosX+i\frac{\gamma }{2}\frac{sinX}{X} \end{array}\right|$$

and

(14) $$X=\sqrt{\phi^{2}+{\left(\frac{\Gamma }{2}\right)}^{2}}$$

in which *M* is the Jones matrix of the liquid crystal layer, *M_T_* is the transpose of *M* upon the reflection, *α* represents the angle between the transmission axis of the polarizer and the rubbing direction of the liquid crystal cell, *ϕ* is total twist angle, and *Γ* is the phase retardation of liquid crystal cell.

**Figure 6:**
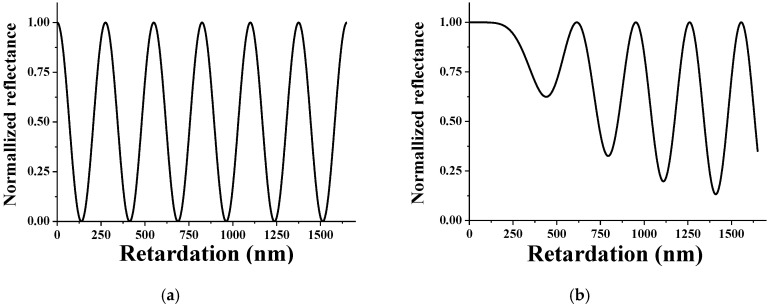
Calculated reflectance of (**a**) splay state and (**b**) π twist state with respect to the retardation.

The reflectance of the splay state and the π twist state is shown in Figure 6. In the splay state, the periodic reflectance value is obtained according to the retardation value, and it is confirmed that the dark state can be realized. As shown in Figure 6b, it can be seen that the reflectance of the twist state fluctuates periodically according to the phase retardation value, but it is impossible to realize a dark state with zero reflectance. Therefore, in order to achieve a high contrast ratio, the splay state must be set to a dark state and the twist state to a bright state.

### 3.3. Contrast Ratio

**Figure 7:**
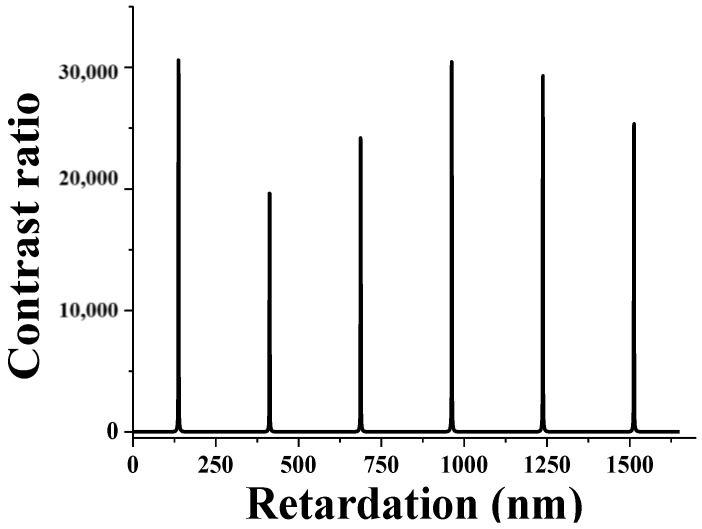
Contrast ratio calculated with respect to the retardation. When the splay state is set to a dark state, a high contrast ratio of about 300,000:1 can be realized.

Figure 7 shows the contrast ratio with respect to the phase retardation. As mentioned above, when the splay state is set to a dark state, a high contrast ratio of about 300,000:1 can be realized. The maximum contrast ratio can be obtained with the phase retardation of 137.5 nm, 412.5 nm and 687.5 nm under the following condition

(15) $$CR_{max}=\;\frac{\lambda }{4}+\frac{m\lambda }{2}$$

## 4. Fabrication of Reflective BCSN

**Figure 8:**
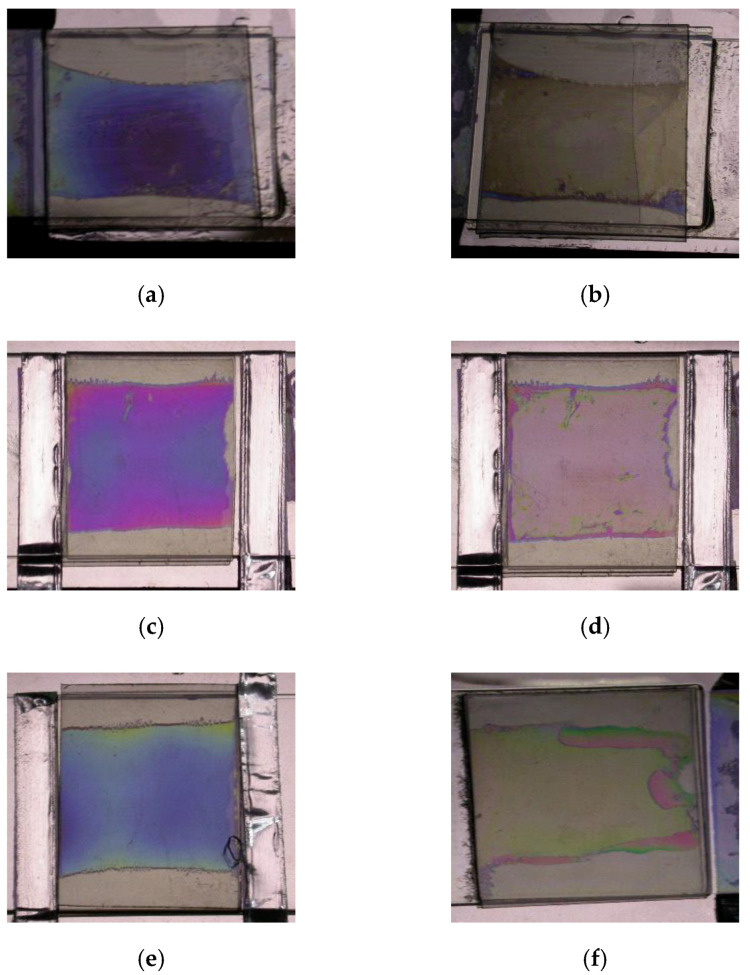
Fabricated BCSN devices with various retardation values. (**a**) splay state of 137.5 nm, (**b**) twist state of 137.5 nm, (**c**) splay state of 412.5 nm, (**d**) twist state of 412.5 nm, (**e**) splay state of 687.5 nm, and (**f**) twist state of 687.5 nm.

**Figure 9:**
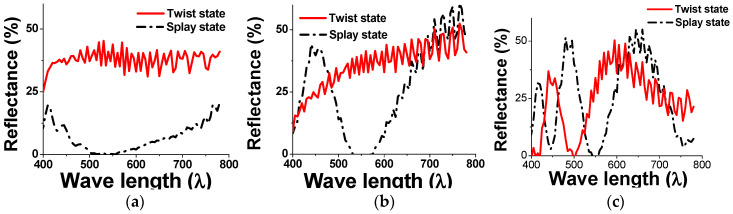
Measured reflectance of both splay and twist states of BCSN device with retardations of (**a**) 137.5 nm, (**b**) 412.5 nm, and (**c**) 687.5 nm.

Several test cells are fabricated to investigate the optical characteristics for the reflective BCSN structure. The substrates are coated with an alignment material AL-90101 (JALS) that produces a pretilt angle of 5° after the rubbing process. A chiral additive material is doped into the host liquid crystal ZLI-2471 (Merck) to yield the *d*/*p* ratio of 0.2. The splay and twist states of fabricated BCSN devices with various retardation values are shown in Figure 8. The measured reflectance with respect to the wavelength of both splay (dark) and twist (bright) states of the fabricated BCSN device is shown in Figure 9. At the 550 nm, the measured contrast ratios are 215, 390, and 330 for the retardations of 137.5 nm, 412.5 nm, and 687.5 nm, respectively.

## 5. Conclusions

The splay state and π twist states are used for the memory states of the BCSN as the low-power heat sensor. With the *d*/*p* ratio of 0.2, the π twist state is replaced by the splay state accompanying the motion of the disclination line starting from the defects or pixel boundaries. The transition status is dependent on the time and temperature due to the temperature dependence of the rotational viscosity value. That is, when the π twist state is exposed to heat at a certain temperature for a certain time, the velocity of transition into the splay state increases. For the heat sensor application, the reflective structure of the BCSN mode is optimized by the Jones matrix method. In experiments, the measured contrast ratio can be over 200 at 550 nm with a minimal reflective structure consisting of a single polarizer and a reflector.
